# Methamphetamine and Modulation Functionality of the Prelimbic Cortex for Developing a Possible Treatment of Alzheimer’s Disease in an Animal Model

**DOI:** 10.3389/fnagi.2021.751913

**Published:** 2021-10-20

**Authors:** Bai-Chuang Shyu, Zhi-Yue Gao, José Jiun-Shian Wu, Alan Bo Han He, Cai-N Cheng, Andrew Chih Wei Huang

**Affiliations:** ^1^Institute of Biomedical Sciences, Academia Sinica, Taipei, Taiwan; ^2^Yuanshan Branch, Taipei Veterans General Hospital, Taipei, Taiwan; ^3^Department of Biology, National Tsing Hua University, Hsinchu, Taiwan; ^4^Department of Psychology, Fo Guang University, Yilan, Taiwan; ^5^Department of Life Sciences, National Central University, Taoyuan City, Taiwan

**Keywords:** methamphetamine (MA), conditioned taste aversion (CTA), prelimbic cortex (PrL), corticosterone (CORT), Alzeheimer’s disease, rats

## Abstract

Alzheimer’s disease (AD) is a progressive neurodegenerative condition that causes cognitive impairment and other neuropsychiatric symptoms. Previously, little research has thus far investigated whether methamphetamine (MAMPH) can enhance cognitive function or ameliorate AD symptoms. This study examined whether a low dose of MAMPH can induce conditioned taste aversion (CTA) learning, or can increase plasma corticosterone levels, neural activity, and neural plasticity in the medial prefrontal cortex (mPFC) (responsible for cognitive function), the nucleus accumbens (NAc) and the amygdala (related to rewarding and aversive emotion), and the hippocampus (responsible for spatial learning). Furthermore, the excitations or lesions of the prelimbic cortex (PrL) can affect MAMPH-induced CTA learning, plasma corticosterone levels, and neural activity or plasticity in the mPFC [i.e., PrL, infralimbic cortex (IL), cingulate cortex 1 (Cg1)], the NAc, the amygdala [i.e., basolateral amygdala (BLA) and central amygdala (CeA)], and the hippocampus [i.e., CA1, CA2, CA3, and dentate gyrus (DG)]. In the experimental procedure, the rats were administered either saline or NMDA solutions, which were injected into the PrL to excite or destroy PrL neurons. Additionally, rats received 0.1% saccharin solution for 15 min, followed by intraperitoneal injections of either normal saline or 1 mg/kg MAMPH to induce CTA. A one-way ANOVA was performed to analyze the effects of saccharin intake on CTA, plasma corticosterone levels, and the expression of c-Fos and p-ERK. The results showed that the MAMPH induced CTA learning and increased plasma corticosterone levels. The mPFC, and particularly the PrL and IL and the DG of the hippocampus, appeared to show increased neural activity in c-Fos expression or neural plasticity in p-ERK expression. The excitation of the PrL neurons upregulated neural activity in c-Fos expression and neural plasticity in p-ERK expression in the PrL and IL. In summary, MAMPH may be able to improve cognitive and executive function in the brain and reduce AD symptoms. Moreover, the excitatory modulation of the PrL with MAMPH administration can facilitate MAMPH-induced neural activity and plasticity in the PrL and IL of the mPFC. The present data provide clinical implications for developing a possible treatment for AD in an animal model.

## Introduction

Previous studies of methamphetamine (MAMPH) have demonstrated that MAMPH is an addictive drug that induces compulsion, craving, and relapsing behaviors (Kirkpatrick et al., 2012; Mizoguchi and Yamada, 2019). Additionally, chronic MAMPH exposure can cause neurotoxicity, neuroinflammation, and neuronal apoptosis in the central nervous system (Shukla and Vincent, 2020). In this context, MAMPH has led to cognitive impairments and premature brain aging caused by Alzheimer’s disease (AD) (Hart et al., 2012; Panenka et al., 2013). However, MAMPH has been beneficial in treating conditions including obesity (Bray, 1993), bipolar depression (Perugi et al., 2017), attention-deficit/hyperactivity disorder (Ramirez et al., 2018), and narcolepsy (Fry, 1998). A fewer research has shown that MAMPH administration may ameliorate some of the behavioral and neuropsychiatric symptoms of AD and could suppress AD-associated proteins and molecular in neurons (Shukla and Vincent, 2020). For example, MAMPH, and a cognitive enhancer, FR121196, boosted memory retention in rats that undertook passive avoidance tasks (Matsuoka et al., 1992). An *in vitro* study demonstrated that the application of MAMPH to the human neuroblastoma SH-SY5Y cell line modulated beta-amyloid precursor protein-cleaving secretases, indicating it may ameliorate cognitive function in AD patients (Shukla et al., 2019). Additionally, long-term, low-dose MAMPH treatments to the dentate granule neuron of the hippocampus slice have facilitated long-term potentiation (LTP) and spinal enlargement (Baptista et al., 2016). Depending on the clinical findings in human models, the administration of psychostimulants (e.g., methylphenidate and amphetamine) could improve the apathy symptom in AD patients (Scherer et al., 2018). Additionally, a one-off, low dose of MAMPH (i.e., 30 mg) appeared to enhance the abilities in attention, information processing speed, and working memory in relation to cognitive performance (Mahoney et al., 2011). Therefore, the low dose of MAMPH may improve behavioral cognitive function, and neuronal activity, or synaptic plasticity.

MAMPH activates different functions in a variety of brain areas (Hart et al., 2012; Shukla and Vincent, 2020). For example, the medial prefrontal cortex (mPFC) comprises the medial (frontal) agranular cortex, the anterior cingulate cortex, the prelimbic cortex (PrL), and the infralimbic cortex (IL) (Morales et al., 2007). It also mediates numerous cognitive functions such as inhibition control, attention, habit formation, spatial learning, working memory, and long-term memory (Riga et al., 2014; Jobson et al., 2021). Regarding aging and dementia, the mPFC is involved in cognitive decline and impairments in cognitive performance (Jobson et al., 2021). In addition, the dense interactivity of the mPFC links to the subcortical regions, including the thalamus, NAc, amygdala, and hippocampus (Riga et al., 2014; Jobson et al., 2021); it also controls certain executive and cognitive functions in these subcortical regions (Dalley et al., 2004). The cingulate cortex area 1 (Cg1) of the mPFC is involved in pain (Vogt and Sikes, 2000), anxiety, and aversion (Singewald et al., 2003). The PrL and IL contribute to MAMPH addiction, which is also associated with conditioned learning (He et al., 2019). Furthermore, the PrL modulates enhancements in aversive stimuli in fear behavior and in rewarding stimuli in drug addiction behavior; conversely, the IL regulates inhibition in fear and facilitation in drug addiction (Peters et al., 2009). Another projected pathway is from the mPFC to the affected brain areas that involve the NAc, which mediates the reward and reinforcement effects of MAMPH addiction (Sun et al., 2013; Cheng et al., 2020). The amygdala was separated into the central amygdala (CeA), which mediates negative emotional responses, and the basolateral amygdala (BLA), which controls the processing of rewarding and aversive stimuli (Tanimoto et al., 2003; Beyeler et al., 2018; Cai et al., 2018). The hippocampus also receives the convey projections from the mPFC and regulates spatial and contextual learning and memory from the subareas of the hippocampus, including the CA1, CA2, CA3, and dentate gyrus (DG) (Jarrard, 1993). Moreover, the hippocampus has been intensively studied in relation to age-related neurological condition, including Alzheimer’s disease (Patrylo and Williamson, 2007). Primarily, the DG mediates spatially related information processing as well as representations of spatial memory retention based on the DG’s conjunctive encoding and spatial pattern separation, and the encoding of spatial information conveyed to the CA3 (Kesner, 2007). Therefore, the present study tested whether the MAMPH administrations stimulated neuronal activity in brain areas that mediate cognition, rewarding and aversive emotion, and spatial learning and memory.

Previous studies of AD animal models have demonstrated that age is a crucial factor in learning and memory (El Tamer et al., 1992, 1996; Gilbert et al., 2009). For example, in one AD animal model, the intracerebroventricular administration of the cholinotoxic ethylcholine aziridinium (AF64A) resulted in reduced choline acetyltransferase (ChAT) activity and increased acetylcholinesterase (AChE) in the hippocampus of 4- and 12-month-old rats, but no effects were observed in 22-month-old rats, which indicated age-dependent effects on AF64A-mediated cholinotoxicity (El Tamer et al., 1996). A similar study using rats aged 1.5–2 months old (i.e., weighing 250–350 g) showed similar results (El Tamer et al., 1992). Another study examining rats aged 6 and 24 months showed similar performances for both age groups in olfactory and visual discrimination tasks; however, the 24-month-old rats displayed worse performance in reversal learning and contextual learning tasks than the 6-month-old rats (Gilbert et al., 2009). Therefore, younger rats may be more appropriate when measuring performance on learning and memory tasks. Therefore, the present study used younger rats, aged 1.5–2 months (6–8 weeks) to test the effects of MAMPH on conditioned taste aversion (CTA).

Regarding previous neuroendocrinological data, plasma corticosterone levels secreted from the hypothalamus-pituitary gland-adrenal gland system can serve as stress biomarkers (Piazza et al., 1991; Harvey et al., 2006). Therefore, this study also investigated whether MAMPH administrations elevate plasma corticosterone levels.

Overall, the present study set out to determine the following:

(a) Whether MAMPH administrations induce conditioned learning in the CTA task, increase plasma corticosterone levels, and neural activity (labeling c-Fos expression) or neural plasticity (labeling p-ERK expression) in the Cg1, PrL, and IL of the mPFC; the NAc; the CeA and BLA of the amygdala; and the CA1, CA2, CA3, and DG of the hippocampus.
(b) Whether microinjecting PrL neurons with low or high concentrations of NMDA modulates MAMPH-induced CTA learning, plasma corticosterone levels, and neural activity or neural plasticity in the mPFC (i.e., Cg1, PrL, and IL), NAc, amygdala (i.e., CeA and BLA), and hippocampus (i.e., CA1, CA2, CA3, and DG).

## Materials and Methods

### Animals

All of the 89 male Wistar rats were obtained from BioLasco Taiwan Co., Ltd. At the commencement of the experiment, each rat weighed between 220 and 330 g. The rats were grouped-housed in the colony rooms with two rats in the plastic cage (47 × 26 × 21 cm) containing hardwood laboratory bedding. The colony room was kept at a constant temperature (approximately 23°C ± 2°C) with a 12 h:12 h light/dark cycle (lights on 0600–1800) maintained throughout the experiment. Water and food were freely available, except for rats on the water deprivation regimen who were deprived of water for 23.5 h/day from the water deprivation to the conditioning phase. The experiment was performed in accordance with the Animal Scientific Procedures Act of 1986 and received approval from the Fo Guang University Institutional Animal Care and Use Committee. The number of animals was limited, and every effort was made to minimize animal suffering during the experiment.

### Apparatus

Lickometer was used to measure the intake volume of water or saccharin solution in the study. The lickometer device comprises a wire-mesh cage, a white panel, and a 25-ml burette with 0.1-ml graduations. The burette was mounted in front of the wire-mesh cage inserted through a hole in the white panel. The intake volume of the saccharin solution served to determine the magnitude of the effect of MAMPH on CTA learning.

### Microinjection Parameters and Experimental Surgery

**FIGURE 1:**
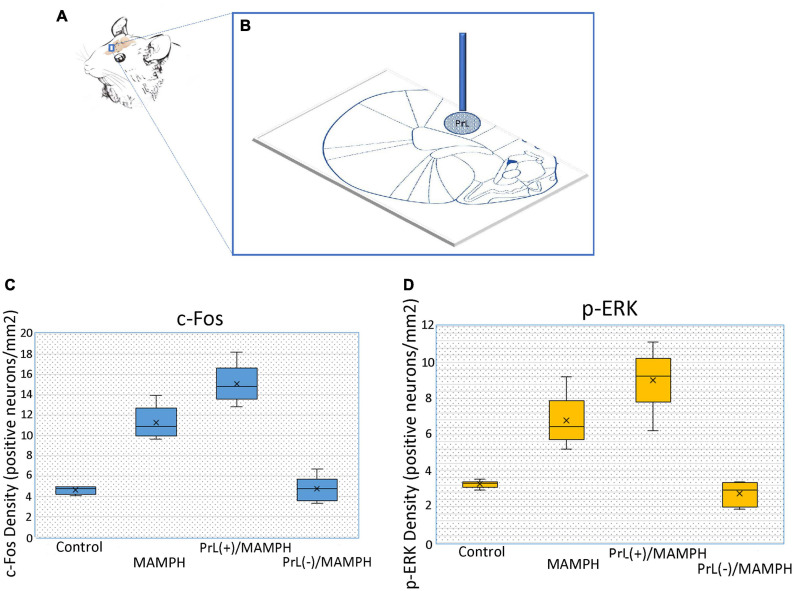
**(A)** The rat’s brain atlas showing the PrL location as a small vertical bar. **(B)** The location of the PrL microinjection. The blue vertical bar represents the buried cannula placement. The spot area indicates the distributions of saline or the NMDA microinjections. The density of **(C)** c-Fos or **(D)** p-ERK in positive neurons/mm^2^ in the PrL for the control, MAMPH, PrL (+)/MAMPH, and PrL(–)/MAMPH groups.

For the surgical procedures, a 1-μl volume of either NMDA or saline was left unilaterally injected into the PrL (anterior-posterior: 3.24 mm from bregma; lateral: 0.60 mm from the midline; dorsal-ventral: 3.00 mm from the skull surface) (Paxinos and Watson, 2007). The substances were injected at a rate of 1 μl/10 min, and the needle was left in the PrL for an additional 10 min. To verify the cannula location, a schematic representation of a left unilateral cannula site was used for reference. The blue dot area indicates a microinjection site of saline or NMDA within the PrL (Figures 1A,B). Figures 1C,D show the density of c-Fos- or p-ERK-positive neurons after the PrL microinjections of saline and low or high concentrations of NMDA solutions before the MAMPH intraperitoneal administrations for the control, MAMPH, PrL(+)/MAMPH, and PrL(–)/MAMPH groups. MAMPH enhanced the density of c-Fos and p-ERK expression in the PrL (*p* < 0.05). The microinjections of low-concentration NMDA significantly increased the density of c-Fos and p-ERK expression compared with the PrL(+)/MAMPH and MAMPH groups (*p* < 0.05). The high concentration of NMDA microinjections in the PrL significantly decreased the density of c-Fos and p-ERK expression compared with the PrL(–)/MAMPH and MAMPH groups (*p* < 0.05; Figure 1).

### Behavioral Procedure

During the 7-day adaptation phase, the rats were allowed to drink water and eat food freely in their home cage. On Day 0, all rats received anesthesia and surgery. Twenty minutes before anesthesia by sodium pentobarbital injection (50 mg/kg, i.p.), the rats were injected with gentamicin (40 mg/kg, i.p.) and atropine sulfate (0.1 mg, i.p.). After the surgery, the outer cannula was kept in the PrL for the later microinjections of saline or low or high concentrations of NMDA to destroy or excite the PrL neurons. All the rats were then allowed to recover in the home cage for 7 days, with food and water freely available (Days 1–7). Next, the rats were deprived of water for 23.5 h/day for 5 days (Days 8–12). On Day 13, the rats were given microinjections of saline and either a low or high concentration of NMDA into the PrL. Later, the rats were subject to CTA conditioning learning for three trials, which was 0.1% saccharin solution for 15 min and then intraperitoneal injections of 1 mg/kg MAMPH or saline to form CTA learning. During the conditioning phase (Days 13–15), the rats underwent another water deprivation regimen where they were allowed to drink a 0.1% saccharin solution for 15 min from the lickometer device in the morning and then water for 30 min in the home cage in the evening. The rats were then separated into control (*n* = 19), MAMPH (*n* = 14), PrL(+)/MAMPH (*n* = 12), and PrL(–)/MAMPH (*n* = 20) groups. The control group was given microinjections of saline into the PrL and intraperitoneal injections of saline in conditioning; the MAMPH group were given microinjections of saline into the PrL and intraperitoneal injections of MAMPH in conditioning; the PrL(+)/MAMPH group were given microinjections of low concentrations of NMDA into the PrL and intraperitoneal injections of MAMPH in conditioning; and the PrL(–)/MAMPH group were given microinjections of high concentrations of NMDA into the PrL and intraperitoneal injections of MAMPH in conditioning.

Ninety minutes after completing the final trial of the CTA learning test, the rats were humanely sacrificed, and their brains were removed for immunohistochemical staining with c-Fos or p-ERK expression. Additionally, blood was collected to assess plasma corticosterone levels using the ELISA method for baseline (on Day 0) and testing (on Day 15; Supplementary Figure 1).

### Immunohistochemical Staining

To begin, the rats were sacrificed via a sodium pentobarbital overdose. Later, the rats were conducted using perfusion with 100 ml of 0.1 M sodium phosphate-buffered saline (PBS) followed by 400 ml of 4% paraformaldehyde in a 0.1 M PBS buffer. Next, the brain tissues were dissected and transferred to a 30% sucrose dissolved in a 4% paraformaldehyde solution for cryoprotection. Once the brain tissues sank to the bottom of the solution, the tissues were then frozen with Tissue-Tek^®^ O.C.T. Compound. Next, 40–μm slices of the brain were obtained using a freezing microtome. The slices were labeled using the immunohistochemical staining for c-Fos and p-ERK expression. These free-floating brain slices were then washed for 15 min in 0.1 M PBS, permeabilized in 3% H_2_O_2_ for 1 h, washed for 20 min three times in 2% PBS tween-20 (PBST), and soaked in 3% normal goat serum and 1% bovine serum albumin for 1 h. Later, all brain slices were washed twice with PBST for 15 min. The slices were incubated at 4°C overnight to carry out c-Fos labeling with primary rabbit anti-Fos antibody (SC-52, 1:1,000; Santa Cruz Biotechnology Inc.) and p-ERK labeling with primary rabbit anti-p-ERK (GTX50274, 1:500; Genetex Inc.). The slices were then washed twice for 10 min with PBST before being incubated with a biotinylated goat anti-rabbit secondary antibody (1:500; Vector Lab BA-1,000) for 1 h. Later, the brain slices were washed for 10 min with PBS, and the ABC kit (Vector Lab ABC Kit, PK-6100) was used to amplify the bound secondary antibody of the brain slices. The positive expression of c-Fos or p-ERK was counted for the entire brain using ImageJ software. Counting was performed visually at 20x magnification for each brain slice. The c-Fos- or p-ERK density positive neurons were analyzed using the formula: c-Fos or p-ERK numbers/the slice areas (0.88 mm × 0.69 mm ≒ 60.72 mm^2^).

### Drugs

The MAMPH was obtained from the Food and Drug Administration, Ministry of Health and Welfare, Executive Yuan (Taipei, Taiwan), while the NMDA and sodium chloride were obtained from Sigma-Aldrich (St. Louis, MO). The MAMPH was dissolved in normal saline to a concentration of 1 mg/ml. The sodium saccharin was dissolved in distilled water and prepared in a 0.1% saccharin solution. The saline and MAMPH were injected intraperitoneally at a volume of 1 ml. The study used a low concentration of 2 mg/ml of NMDA and a high concentration of 20 mg/ml, in line with the concentrations suggested by the previous study (Huang et al., 2020).

### Statistical Analysis

A one-way ANOVA was performed to analyze the volume of saccharin solution intake to measure MAMPH-injected producing the CTA learning for the control, MAMPH, PrL (+)/MAMPH, PrL(–)/MAMPH groups over the first three sessions. Furthermore, the effect size (i.e., partial η^2^) and the power value were performed. When appropriate, Tukey’s honestly significant difference (HSD) *post hoc* test was performed for each session. A *p*-value of < 0.05 was considered significant in all cases. A one-way ANOVA was performed for plasma corticosterone levels and the immunohistochemical staining data for c-Fos and p-ERK expression in the selected brain areas for the control, MAMPH, PrL (+)/MAMPH, and PrL(–)/MAMPH groups at baseline and in the final conditioning (testing phase). Furthermore, the effect size (i.e., partial η^2^) and the power value were performed. When appropriate, Tukey’s HSD *post hoc* test was conducted for each session. A *p*-value of < 0.05 was considered significant in all cases.

Partialη^2^ is the ratio of variance related to an effect, plus that effect and its associated error variance. The formula was to calculate partialη^2^ as follows:

Partialη^2^ = SSeffect/(SSeffect + SSerror)

The following rules of thumb are applied to interpret values for partialη^2^: 0.01: Small effect size; 0.06: Medium effect size; 0.14 or higher: Large effect size.

Power values are the probability of correctly rejecting a null hypothesis (H0) when it is false. The formula was to calculate power as follows: Power = *P*(reject H0/H0 is false). A statistical power value is of 0.80 or higher, and the experimental results lead to valid conclusions about the meaning of the results.

## Results

The study tested MAMPH administrations associated with PrL modulation over three sessions once a session for the conditioned learning, plasma corticosterone levels, the c-Fos or p-ERK expression for the mPFC, the amygdala, the NAc, and the hippocampus as follows.

**FIGURE 2:**
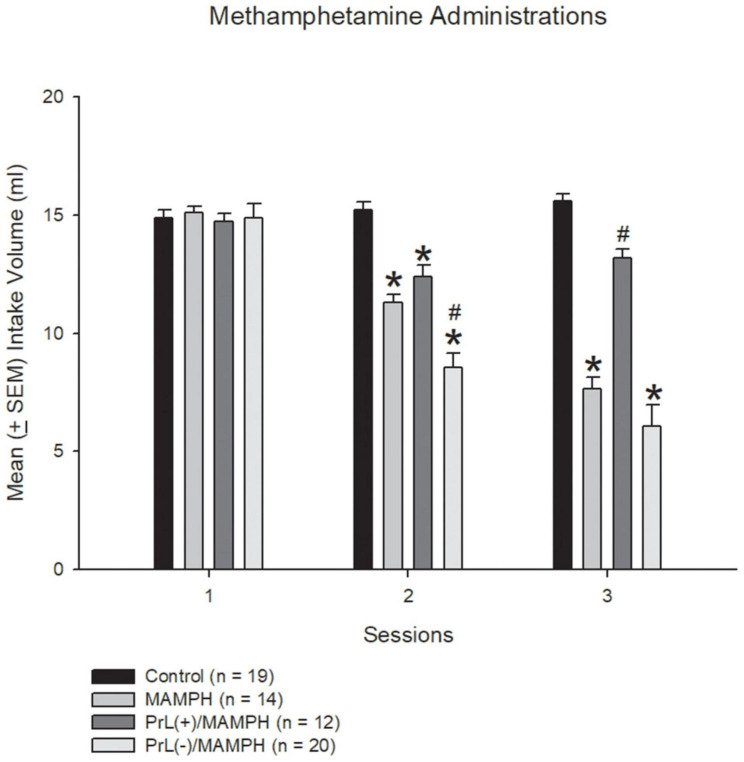
MAMPH-induced conditioned taste aversion tested for behavior using NMDA or saline injections. Mean (± SEM) intake volume of 0.1% saccharin solution for three sessions (with 1 mg/kg of intraperitoneally injected MAMPH) across the control (*n* = 19), MAMPH (*n* = 14), PrL (+)/MAMPH (*n* = 12), and PrL(–)/MAMPH groups (*n* = 20). PrL(+): Injection of a low concentration of NMDA to excite the PrL neurons; PrL(–): Injection of a high concentration of NMDA to destroy the PrL neurons. Differences in the intake volumes of 0.1% saccharin solution among all groups in each session were analyzed by one-way ANOVA, followed by Tukey’s *post hoc* test if a significant difference was appeared among groups. *p < 0.05 indicates significant differences compared with the control group; ^#^*p* < 0.05 indicates significant differences compared with the MAMPH group.

In the beginning, we tested the MAMPH administrations induced CTA conditioned learning and the excitation or the lesion of the PrL underlying MAMPH-induced CTA learning. One-way ANOVA was analyzed showed that the factor of group showed significant differences in session 2 [*F*(3, 61) = 40.20, *p* < 0.05] (partial η^2^ = 0.64, power = 1.00) and in session 3 [*F*(3, 61) = 53.00, *p* < 0.05] (partial η^2^ = 0.72, power = 1.00), but not a significant difference in session 1 [*F*(3, 61) = 0.90, *p* > 0.05] (partial η^2^ = 0.00, power = 0.07). The *post hoc* Tukey’s test showed that the saccharin intake volumes of the MAMPH, PrL(+)/MAMPH, and PrL(–)/MAMPH groups during session 2 were significantly reduced compared with the control group (*p* < 0.05). The intake volume of the PrL(–)/MAMPH group was significantly less than that of the MAMPH group (*p* < 0.05). In session 3, the MAMPH and PrL(–)/MAMPH groups showed significantly reduced intake volumes of the saccharin solution; however, the PrL(+)/MAMPH group showed a significant increase in intake volume compared with the MAMPH group. Therefore, we can conclude that MAMPH administration in session 1 did not induce CTA conditioned learning. However, the MAMPH injections in sessions 2 and 3 reduced the saccharin solution intake enough to induce CTA conditioned learning. The high concentrations of NMDA microinjected into the PrL destroyed the PrL neurons. The lesion of the PrL neurons enhanced the MAMPH-induced CTA conditioned learning in session 2. In contrast, the low concentration of NMDA microinjected in the PrL, excited the PrL neurons enough to blunt the MAMPH-induced CTA learning in session 3 (Figure 2).

Regarding the analysis of the plasma corticosterone levels, one-way ANOVA analysis indicated that there were no significant differences between the control, MAMPH, PrL(+)/MAMPH, and PrL(–)/MAMPH groups [*F*(3, 20) = 0.14, *p* > 0.05] (partial η^2^ = 0.02, power = 0.07) in the baseline. During the test phase, significant differences were found in group [*F*(3, 20) = 33.67, *p* < 0.05] (partial η^2^ = 0.84, power = 1.00). The *post hoc* Tukey HSD test indicated that plasma corticosterone levels in the MAMPH and PrL(–)/MAMPH groups had increased significantly compared with the control group (*p* < 0.05), while plasma corticosterone levels in the PrL(+)/MAMPH group were significantly less than those in the MAMPH group (*p* < 0.05).

**FIGURE 3:**
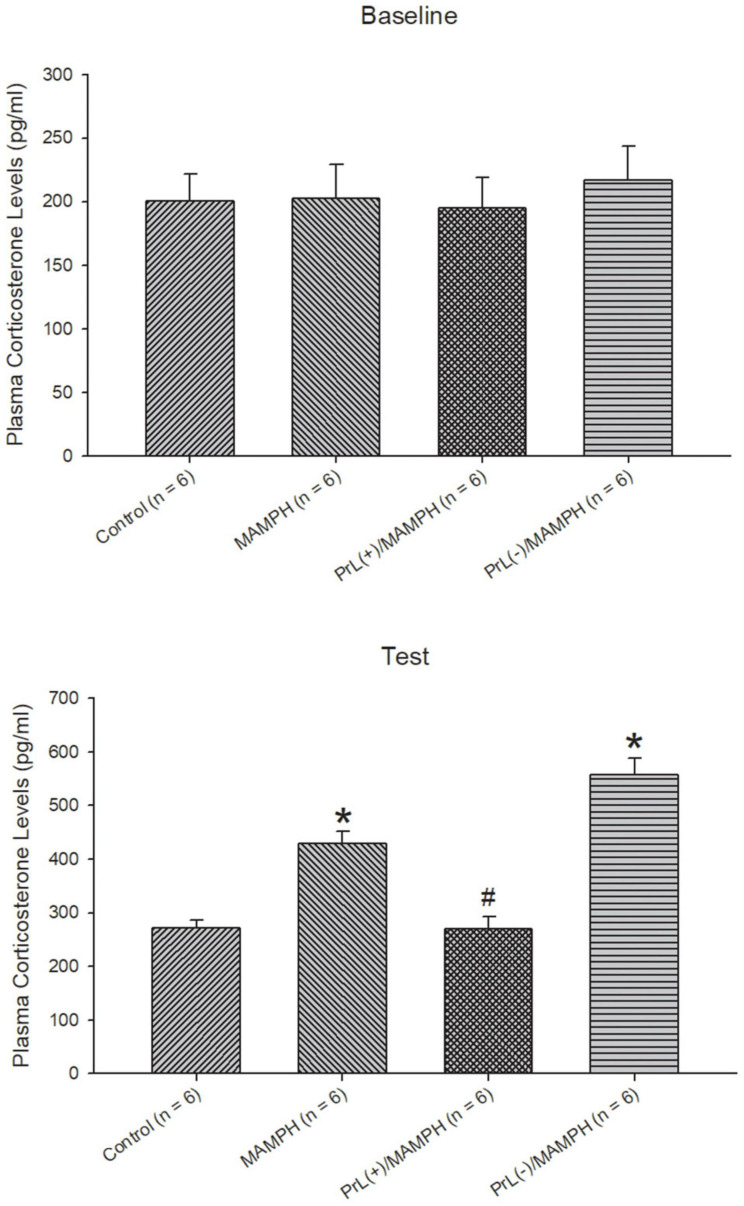
The effect of MAMPH administrations on plasma corticosterone levels were tested using NMDA or saline injections into the PrL. Mean (± SEM) plasma corticosterone levels (pg/ml) were taken for the control (*n* = 6), MAMPH (*n* = 6), PrL(+)/MAMPH (*n* = 6), and PrL(–)/MAMPH groups (*n* = 6) at the baseline and in the conditioning phase. PrL(+): Injection of a low concentration of NMDA to excite the PrL neurons; PrL(–): Injection of a high concentration of NMDA to destroy the PrL neurons. Plasma corticosterone levels were analyzed by one-way ANOVA, followed by Tukey’s *post hoc* test if a significant difference was appeared among groups at the baseline and test phases. ^∗^*p* < 0.05 indicates significant differences compared with the control group; ^#^*p* < 0.05 indicates significant differences compared with the MAMPH group.

In summary, the test phase MAMPH administrations increased plasma corticosterone levels. Moreover, the lesion of the PrL with MAMPH injections enhanced plasma corticosterone levels but exciting the PrL blunted plasma corticosterone levels compared with the single MAMPH administrations (Figure 3).

**FIGURE 4:**
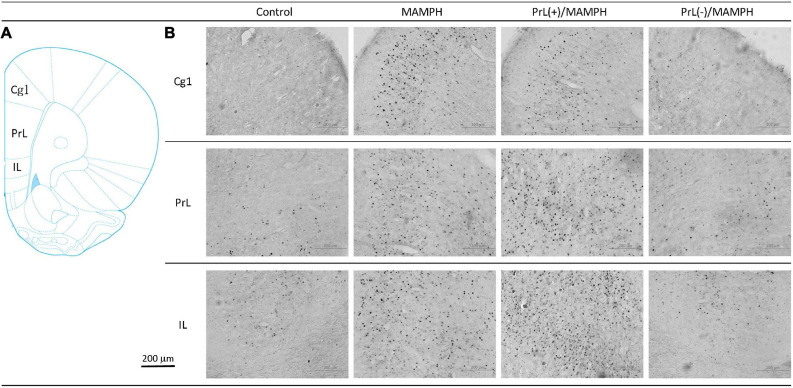
**(A)** A schematic brain atlas for the Cg1, PrL, and IL of the mPFC. **(B)** Representative photomicrographs of c-Fos expression for the Cg1, PrL, and IL in the control, MAMPH, PrL(+)/MAMPH, and PrL(–)/MAMPH groups. The scale bar represents 200 μm.

**FIGURE 5:**
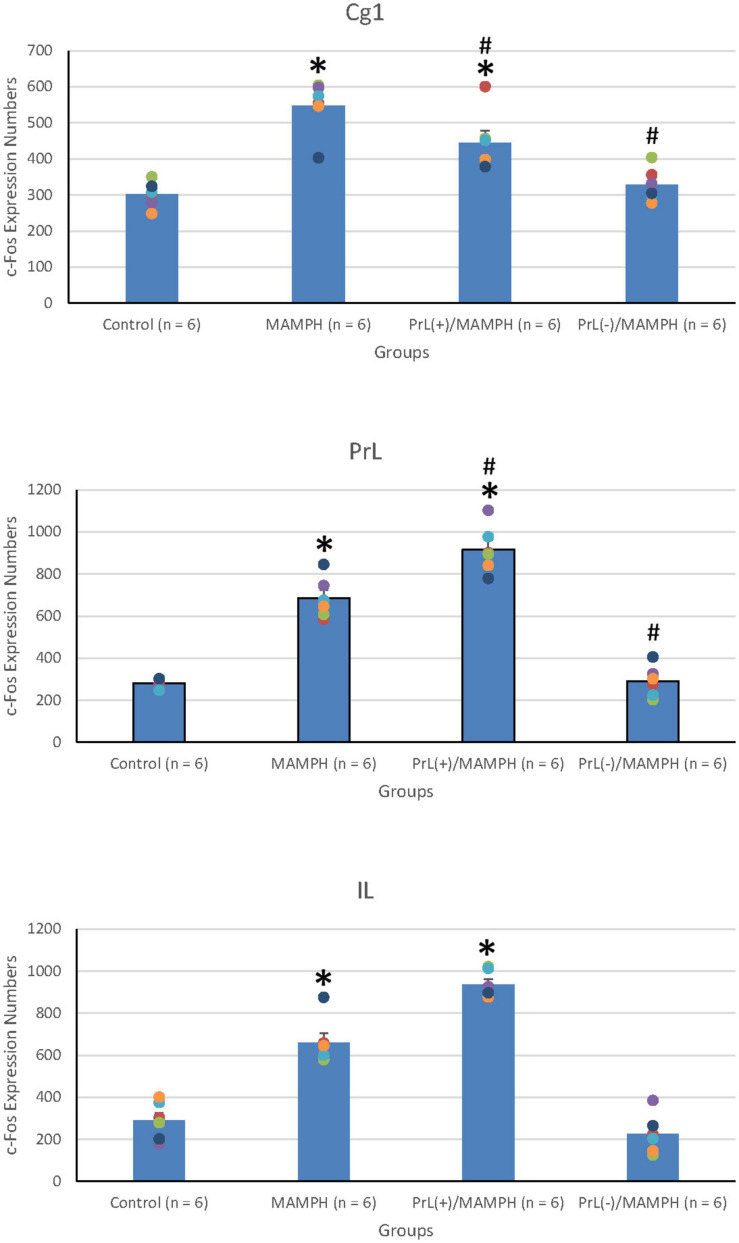
MAMPH administrations were tested to label c-Fos expression in the Cg1, PrL, and IL of the mPFC for the control (*n* = 6), MAMPH (*n* = 6), PrL(+)/MAMPH (*n* = 6), and PrL(–)/MAMPH groups (*n* = 6) by injecting NMDA or saline into the PrL. PrL(+): injection of a low concentration of NMDA to excite the PrL neurons; PrL(–): injection of a high concentration of NMDA to destroy the PrL neurons. Differences in c-Fos expression levels in the Cg1, PrL, and IL among all groups were analyzed by one-way ANOVA, followed by Tukey’s *post hoc* test if a significant difference was appeared among groups. ^∗^*p* < 0.05 indicates significant differences compared with the control group; ^#^*p* < 0.05 indicates significant differences compared with the MAMPH group.

In order to examine the effects of MAMPH administrations with the PrL modulation in cognitive functions using c-Fos expression, a one-way ANOVA was performed, which indicated that the factor of group was the significant difference in the Cg1[*F*(3, 20) = 19.13, *p* < 0.05] (partial η^2^ = 0.74, power = 1.00), PrL [*F*(3, 20) = 83.40, *p* < 0.05] (partial η^2^ = 0.93, power = 1.00), and IL [*F*(3, 20) = 80.02, *p* < 0.05] (partial η^2^ = 0.92, power = 1.00). The subareas of the mPFC, such as the Cg1, PrL, and IL, were identified with neural activity labeling c-Fos expression. The *post hoc* Tukey’s HSD test revealed that the addition of MAMPH significantly increased c-Fos expression compared with the control group for the Cg1, PrL, and IL (*p* < 0.05). Moreover, the PrL(+)/MAMPH group showed a significant increase in c-Fos expression for PrL and IL (p < 0.05). The Cg1 showed a significant decrease in c-Fos expression in the PrL(+)/MAMPH group when compared with the MAMPH group (*p* < 0.05). However, the PrL(–)/MAMPH group was significantly reduced compared to the MAMPH group (*p* < 0.05) in the subareas of the mPFC, including the Cg1, PrL, and the IL (Figures 4, 5). In conclusion, MAMPH administrations may activate neural activity in brain areas associated with cognitive function, including the Cg1, PrL, and IL. The excitation of the PrL neurons increased this neural activity, which was induced by MAMH administration in the PrL and IL. Moreover, the lesion of the PrL neurons blunted MAMPH-induced neural activity in all subareas of the mPFC (i.e., the Cg1, PrL, and IL).

**FIGURE 6:**
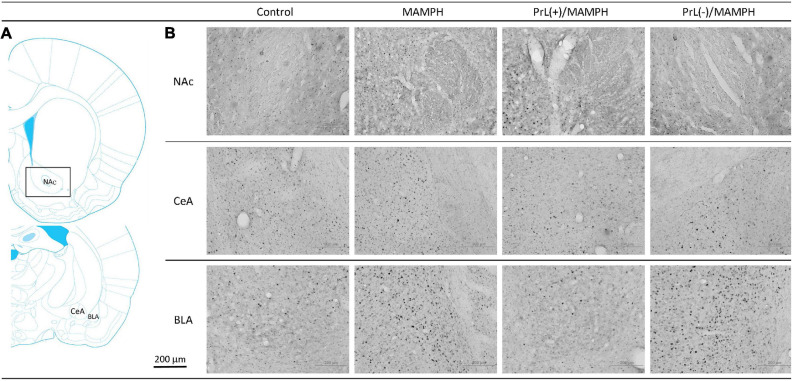
**(A)** A schematic brain atlas for the NAc, and the amygdala’s CeA and BLA. **(B)** Representative photomicrographs of c-Fos expression for the NAc and the amygdala’s CeA and BLA in the control, MAMPH, PrL(+)/MAMPH, and PrL(–)/MAMPH groups. The scale bar represents 200 μm.

**FIGURE 7:**
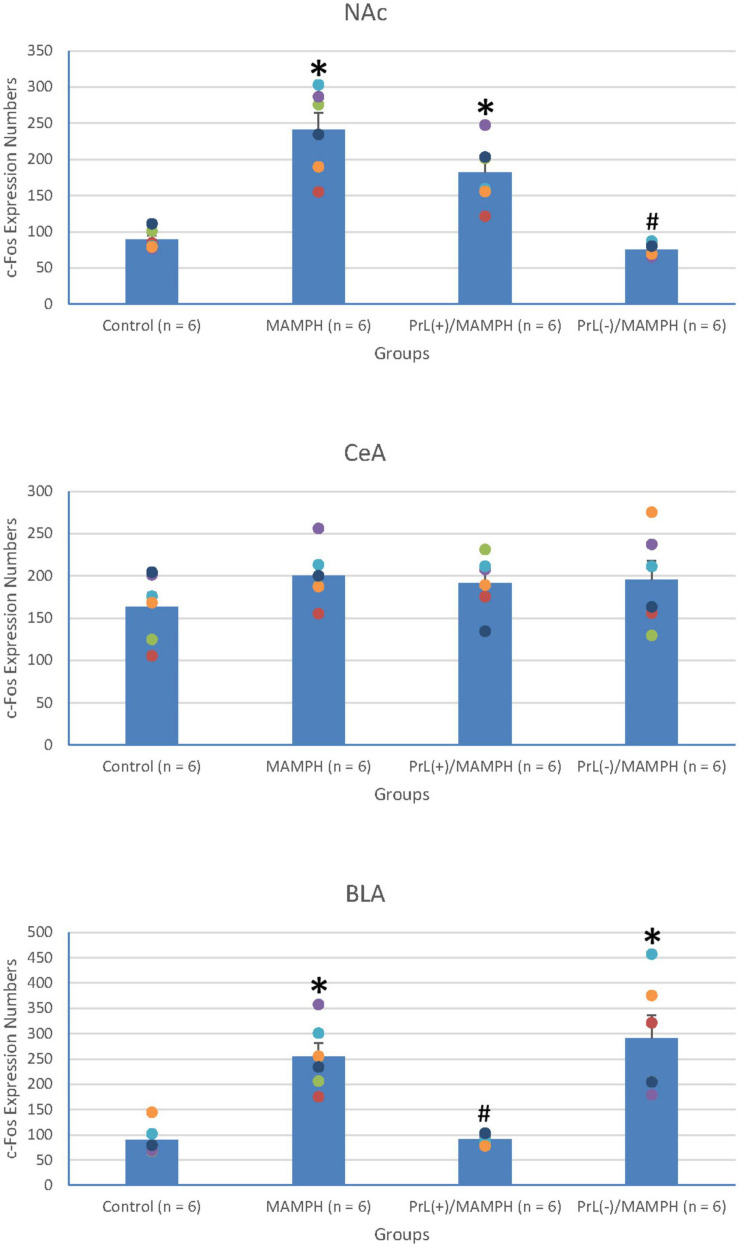
MAMPH administrations were tested to label c-Fos expression in the NAc and the amygdala’s CeA, and BLA for the control (*n* = 6), MAMPH (*n* = 6), PrL(+)/MAMPH (*n* = 6), and PrL(–)/MAMPH groups (*n* = 6) by injecting NMDA or saline into the PrL. PrL(+): Injection of a low concentration of NMDA to excite the PrL neurons; PrL(–): Injection of a high concentration of NMDA to destroy the PrL neurons. Differences in c-Fos expression levels in the NAc, CeA, and BLA among all groups were analyzed by one-way ANOVA, followed by Tukey’s *post hoc* test if a significant difference was appeared among groups. ^∗^*p* < 0.05 indicates significant differences compared with the control group; ^#^*p* < 0.05 indicates significant differences compared with the MAMPH group.

To test MAMPH-induced neural activity and the modulation of the PrL in c-Fos expression, a one-way ANOVA analysis was performed, which showed that the main factor was significant differences in the NAc [*F*(3, 20) = 28.82, *p* < 0.05] (partial η^2^ = 0.80, power = 1.00), CeA [*F*(3, 20) = 0.94, *p* > 0.05] (partial η^2^ = 0.12, power = 0.22), and BLA [*F*(3, 20) = 16.06, *p* < 0.05] (partial η^2^ = 0.69, power = 1.00). The *post hoc* Tukey’s HSD test showed that the MAMPH group significantly increased c-Fos expression in the NAc and BLA but not in the CeA compared with the control group (*p* < 0.05), indicating that MAMPH increased neural activity in the NAc and BLA. In the NAc, c-Fos expression in the PrL(+)/MAMPH group showed no significant differences compared with the MAMPH group (*p* > 0.05). However, the PrL(–)/MAMPH group showed a significant decrease in c-Fos expression compared with the MAMPH group (*p* < 0.05), indicating the lesion of the PrL neurons blocked neural activity in the NAc. In the BLA, the PrL(+)/MAMPH group significantly decreased c-Fos expression compared with the MAMPH group (*p* < 0.05). However, the PrL(–)/MAMPH group did not show any significant differences in c-Fos expression compared with the MAMPH group (*p* > 0.05; Figures 6, 7).

In conclusion, the rewarding and aversive emotion-related brain areas—the NAc and BLA but not the CeA—elicited neural activity following MAMPH administrations. The excitation of the PrL neurons decreased MAMPH-induced neural activity in the BLA. Moreover, the lesion of the PrL neurons blunted MAMPH-elicited neural activity in the NAc.

**FIGURE 8:**
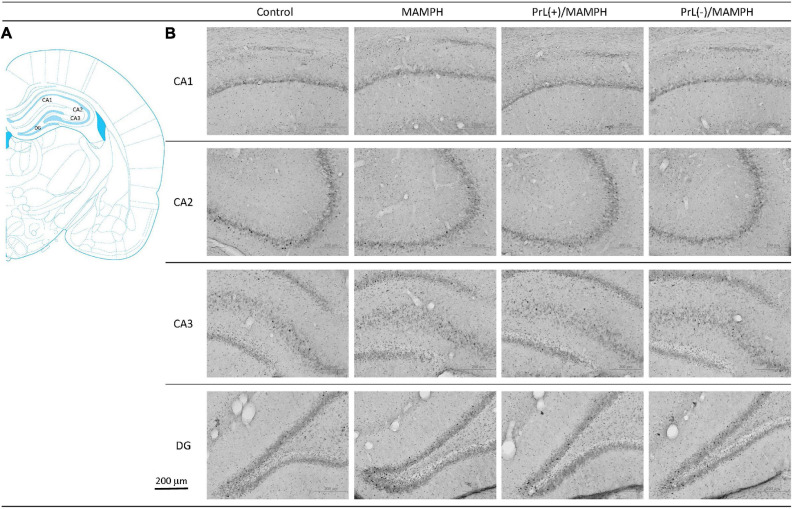
**(A)** A schematic brain atlas for the CA1, CA2, CA3, and DG of the hippocampus. **(B)** Representative photomicrographs of c-Fos expression for the CA1, CA2, CA3, and DG of the hippocampus in the control, MAMPH, PrL(+)/MAMPH, and PrL(–)/MAMPH groups. The scale bar represents 200 μm.

**FIGURE 9:**
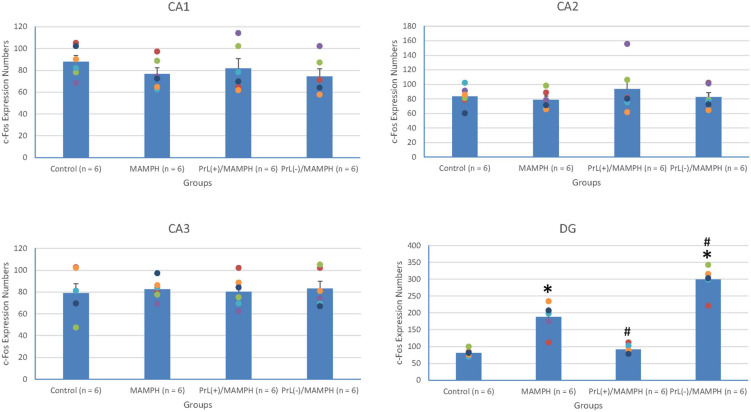
MAMPH administrations were tested in order to label c-Fos expression in the CA1, CA2, CA3, and DG of the hippocampus for the control (*n* = 6), MAMPH (*n* = 6), PrL(+)/MAMPH (*n* = 6), and PrL(–)/MAMPH groups (*n* = 6) by injecting NMDA or saline into the PrL. PrL(+): injection of a low concentration of NMDA to excite the PrL neurons; PrL(–): Injection of a high concentration of NMDA to destroy the PrL neurons. Differences in c-Fos expression levels in the CA1, CA2, CA3, and DG among all groups were analyzed by one-way ANOVA, followed by Tukey’s *post hoc* test if a significant difference was appeared among groups. ^∗^*p* < 0.05 indicates significant differences compared with the control group; ^#^*p* < 0.05 indicates significant differences compared with the MAMPH group.

By analyzing c-Fos expression to elucidate the effect of MAMPH administrations and the modulation of the PrL underlying the MAMPH injections, a one-way ANOVA was conducted, which showed that significant differences occurred in the DG [*F*(3, 20) = 66.26, *p* < 0.05] (partial η^2^ = 0.91, power = 1.00). However, there were no significant differences in the CA1 [*F*(3, 20) = 0.74, *p* > 0.05] (partial η^2^ = 0.10, power = 0.18), CA2 [*F*(3, 20) = 0.56, *p* > 0.05] (partial η^2^ = 0.08, power = 0.15), and CA3 [*F*(3, 20) = 0.10, *p* > 0.05] (partial η^2^ = 0.01, power = 0.06). The *post hoc* Tukey’s HSD test showed that, in the DG, c-Fos expression in the MAMPH and the PrL(–)/MAMPH groups increased significantly compared with the control group (*p* > 0.05). The PrL(–)/MAMPH group showed a significant decrease (*p* < 0.05), while the PrL(+)/MAMPH group showed a significant increase (*p* < 0.05) in the DG’s c-Fos expression (Figures 8, 9). Therefore, it can be surmised that the addition of MAMPH only elicited neural activity in the DG but not in the other subareas of the hippocampus such as the CA1, CA2, and CA3. In the DG, the excitation of the PrL neurons reduced MAMPH administration-induced neural activity, while the lesion of the DG neurons significantly enhanced MAMPH administration-induced neural activity.

In conclusion, the brain areas associated with spatial learning function, the CA1, CA2, CA3, and DG of the hippocampus, were examined by MAMPH administration and PrL’s modulation underlying the MAMPH injections. Only the DG showed neural activity following MAMPH administration. In the DG, the excitation of the PrL neurons blunted MAMPH-induced neural activity, while the lesion of the PrL neurons enhanced MAMPH-induced neural activity.

In order to examine neural plasticity using p-ERK expression labeling in the areas of the brain associated with cognitive function, including the mPFC (e.g., Cg1, PrL, and IL), a one-way ANOVA analysis was performed, which showed that the factor of group was the significant difference in the Cg1 [*F*(3, 20) = 11.23, *p* < 0.05] (partial η^2^ = 0.63, power = 1.00), PrL [*F*(3, 20) = 41.04, *p* < 0.05] (partial η^2^ = 0.86, power = 1.00), and IL [*F*(3, 20) = 35.53, *p* < 0.05] (partial η^2^ = 0.84, power = 1.00).

**FIGURE 10:**
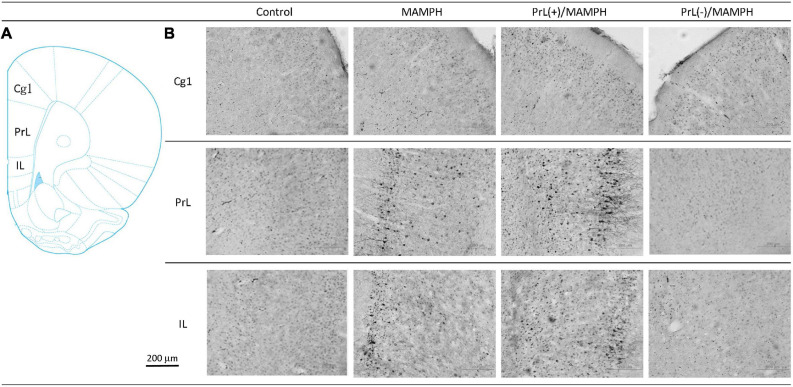
**(A)** A schematic brain atlas for the Cg1, PrL, and IL of the mPFC. **(B)** Representative photomicrographs of p-ERK expression for the Cg1, PrL, and IL in the control, MAMPH, PrL(+)/MAMPH, and PrL(–)/MAMPH groups. The scale bar represents 200 μm.

**FIGURE 11:**
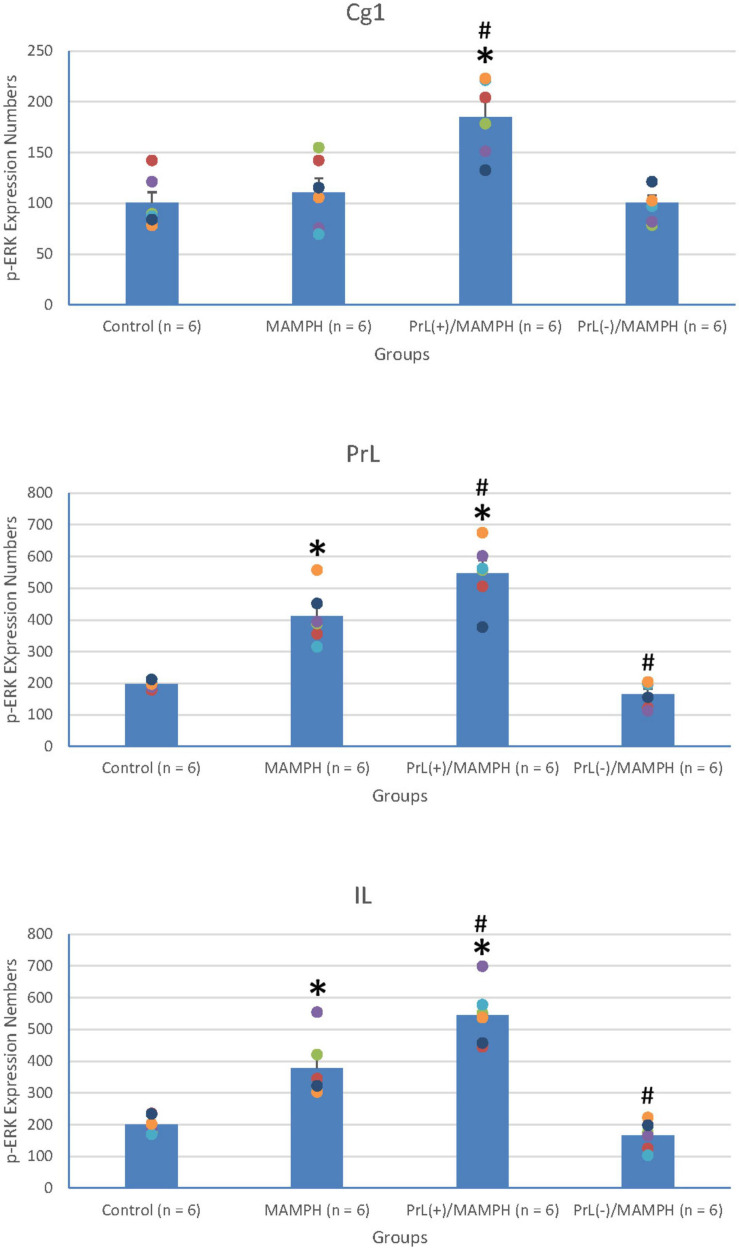
MAMPH administrations were tested to label p-ERK expression in the Cg1, PrL, and IL of the mPFC for the control (*n* = 6), MAMPH (*n* = 6), PrL(+)/MAMPH (*n* = 6), and PrL(–)/MAMPH groups (*n* = 6) by injecting NMDA or saline into the PrL. PrL(+): Injection of a low concentration of NMDA to excite the PrL neurons; PrL(–): Injection of a high concentration of NMDA to destroy the PrL neurons. Differences in p-ERK expression levels in the Cg1, PrL, and IL among all groups were analyzed by one-way ANOVA, followed by Tukey’s *post hoc* test if a significant difference was appeared among groups. ^∗^*p* < 0.05 indicates significant differences compared to the Control group; #*p* < 0.05 indicates significant differences compared to the MAMPH group.

Additionally, the *post hoc* Tukey’s HSD test indicated that p-ERK expression in the MAMPH group increased significantly in the PrL (*p* < 0.05), and IL (*p* < 0.05) compared with the control group, but not in the Cg1 (*p* > 0.05). p-ERK expression increased significantly in the Cg1, PrL, and IL for the PrL(+)/MAMPH group compared with the MAMPH group (*p* < 0.05). In contrast, p-ERK expression in the PrL(–)/MAMPH group decreased significantly in the PrL and BLA compared with the MAMPH group (*p* < 0.05; Figures 10, 11). Therefore, we can surmise that MAMPH elicited neural plasticity in the PrL and IL but not in the Cg1. The excitation of the PrL neurons enhanced MAMPH-induced neural plasticity throughout the entire mPFC, including the Cg1, PrL, and IL. Additionally, the lesion of the PrL neurons reduced MAMPH-induced neural plasticity in the PrL and IL.

**FIGURE 12:**
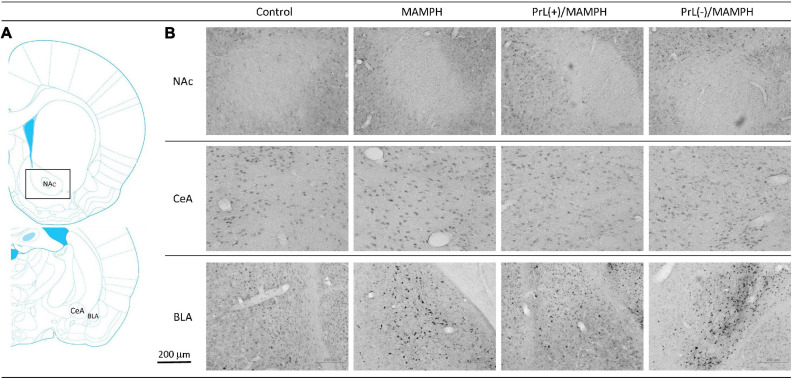
**(A)** A schematic brain atlas for the NAc and the amygdala’s CeA and BLA. **(B)** Representative photomicrographs of p-ERK expression for the NAc and the amygdala’s CeA and BLA in the control, MAMPH, PrL(+)/MAMPH, and PrL(–)/MAMPH groups. The scale bar represents 200 μm.

**FIGURE 13:**
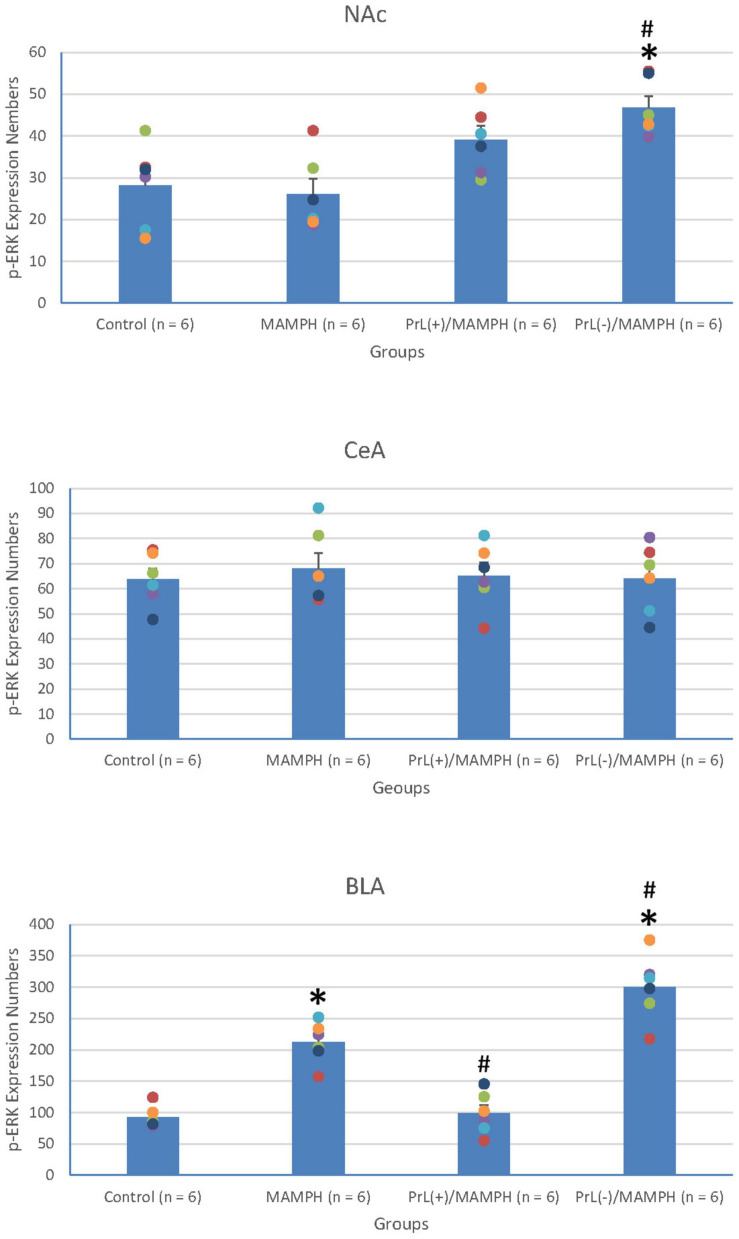
MAMPH administrations were tested in order to label p-ERK expression in the NAc and the amygdala’s CeA, and BLA for the control (*n* = 6), MAMPH (*n* = 6), PrL(+)/MAMPH (*n* = 6), and PrL(–)/MAMPH groups (*n* = 6) by injecting NMDA or saline into the PrL. PrL(+): Injection of a low concentration of NMDA to excite the PrL neurons; PrL(–): Injection of a high concentration of NMDA to destroy the PrL neurons. Differences in p-ERK expression levels in the NAc, CeA, and BLA among all groups were analyzed by one-way ANOVA, followed by Tukey’s *post hoc* test if a significant difference was appeared among groups. ^∗^*p* < 0.05 indicates significant differences compared with the control group; ^#^*p* < 0.05 indicates significant differences compared with the MAMPH group.

To test the effect of MAMPH administrations and the PrL’s modulation underlying the MAMPH injections on p-ERK expression in the rewarding and aversive emotion-related brain areas such as the NAc, CeA, and BLA, a one-way ANOVA analysis showed that the factor of the group was the significant difference in the NAc [*F*(3, 20) = 7.71, *p* < 0.05] (partial η^2^ = 0.54, power = 0.97) and BLA [*F*(3, 20) = 45.19, *p* < 0.05] (partial η^2^ = 0.87, power = 1.00), but not in the CeA [*F*(3, 20) = 0.13, *p* > 0.05] (partial η^2^ = 0.02, power = 0.07). The *post hoc* Tukey’s HSD test indicated that p-ERK expression in the MAMPH group increased significantly compared with the control group in the BLA (*p* < 0.05) but not in the NAc and CeA (*p* > 0.05). In the BLA, p-ERK expression in the PrL(+)/MAMPH group decreased significantly compared with the MAMPH group (*p* < 0.05), while it increased significantly in the PrL(–)/MAMPH group compared with the MAMPH group (*p* < 0.05; Figures 12, 13).

In conclusion, MAMPH elicited neural plasticity in the BLA but not in the CeA and NAc. The excitation of the PrL neurons inhibited MAMPH-induced neural plasticity, while the lesion of the PrL neurons enhanced MAMPH-induced neural plasticity in the BLA.

**FIGURE 14:**
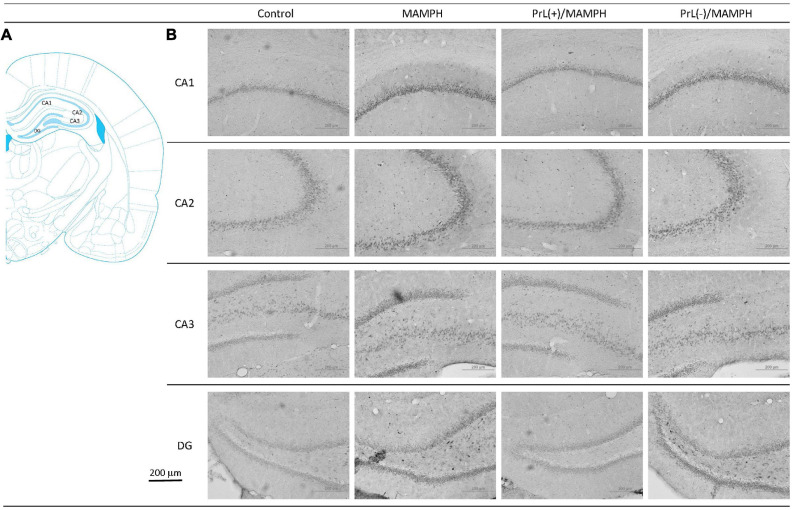
**(A)** A schematic brain atlas for the CA1, CA2, CA3, and DG of the hippocampus. **(B)** Representative photomicrographs of p-ERK expression for the CA1, CA2, CA3, and DG of the hippocampus in the control, MAMPH, PrL(+)/MAMPH, and PrL(–)/MAMPH groups. The scale bar represents 200 μm.

**FIGURE 15:**
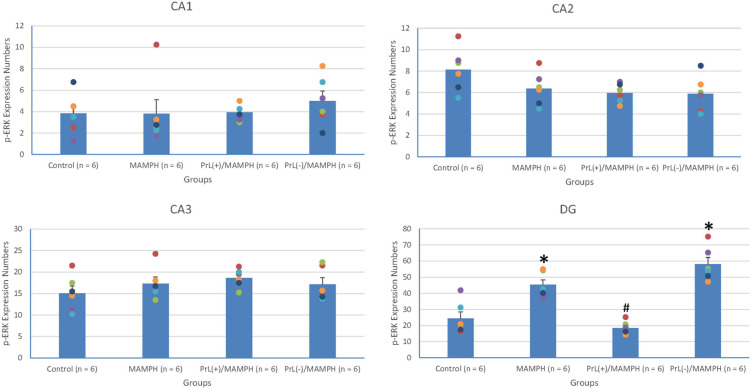
MAMPH administrations were tested to label p-ERK expression in the CA1, CA2, CA3, and DG of the hippocampus for the control (*n* = 6), MAMPH (*n* = 6), PrL(+)/MAMPH (*n* = 6), and PrL(–)/MAMPH groups (*n* = 6) by injecting NMDA or saline into the PrL. PrL(+): Injection of a low concentration of NMDA to excite the PrL neurons; PrL(–): Injection of a high concentration of NMDA to destroy the PrL neurons. Differences in p-ERK expression levels in the CA1, CA2, CA3, and DG among all groups were analyzed by one-way ANOVA, followed by Tukey’s *post hoc* test if a significant difference was appeared among groups. ^∗^*p* < 0.05 indicates significant differences compared with the control group; ^#^*p* < 0.05 indicates significant differences compared with the MAMPH group.

A one-way ANOVA analysis was performed in order to examine MAMPH and the PrL’s modulation underlying the MAMPH administrations. The results of p-ERK expression showed that a factor of group was significant difference in the DG [*F*(3, 20) = 28.49, *p* < 0.05] (partial η^2^ = 0.81, power = 1.00) but not the CA1 [*F*(3, 20) = 0.41, *p* > 0.05] (partial η^2^ = 0.06, power = 0.12), CA2 [*F*(3, 20) = 2.65, *p* > 0.05] (partial η^2^ = 0.28, power = 0.56), and CA3 [*F*(3, 20) = 1.04, *p* > 0.05] (partial η^2^ = 0.14, power = 0.24). The *post hoc* Tukey’s HSD test showed that p-ERK expression in the MAMPH group increased significantly compared with the control group in the DG (*p* < 0.05). In the DG, p-ERK expression in the PrL(+)/MAMPH decreased significantly compared with the MAMPH group (*p* < 0.05). However, there were no significant differences in p-ERK expression between the PrL(–)/MAMPH group and the MAMPH group in the DG (*p* > 0.05; Figures 14, 15). The administration of MAMPH only appeared to induce neural plasticity in the DG. The excitation of the PrL neurons suppressed MAMPH-induced neural plasticity in the DG, while the lesion of the PrL neurons did not affect MAMPH-induced neural plasticity in the DG.

## Discussion

Long-term MAMPH administrations elicited CTA learning and plasma corticosterone levels. At this condition, the brain areas related to cognition, such as the mPFC (e.g., the Cg1, PrL, and IL), the affective areas, such as the rewarding NAc and the aversive and rewarding BLA, and the spatial learning brain area, the DG, activate neural activity for labeling c-Fos expression. Neural plasticity was induced in the PrL, IL, BLA, and DG for labeling p-ERK expression following MAMPH administration. Although microinjections of a low concentration of NMDA enhanced MAMPH-induced neural activity in the brain areas associated with cognitive function (the Cg1, PrL, and IL), the microinjections reduced MAMPH-induced neural activity and plasticity in the BLA and DG. In contrast, microinjections of a high concentration of NMDA reduced MAMPH-induced neural activity in the Cg1, PrL, and IL, and neural plasticity in the PrL and IL, but increased MAMPH-induced neural activity in the DG and neural plasticity in the BLA and DG (Supplementary Table 1).

### Comparison With Previous Findings: Methamphetamine Treatments in Alzheimer’s Disease in the Animal and Human Models

Previous studies have shown that MAMPH administrations can cause neurotoxicity, inflammation, apoptosis, and cell death, or can also produce ameliorative effects in cognitive enhancement and synaptic plasticity, depending on differing doses, and the trials and durations of MAMPH (Hart et al., 2012; Shukla and Vincent, 2020). The low and medium doses or the one-off or short-term MAMPH administrations can ameliorate cognitive decline and dysfunction in AD patients (Hart et al., 2012; Shukla and Vincent, 2020). However, high doses and long-term use of MAMPH are likely to induce neuropsychiatric symptoms (Scherer et al., 2018) and cognitive dysfunction in AD patients (Hart et al., 2012; Shukla and Vincent, 2020).

Regarding neuronal levels, long-term treatments and high doses of MAMPH can cause neuronal inflammation and neurotoxicity (Panenka et al., 2013; Wood et al., 2014). A previous study on people showed that injecting a low dose of 30 mg MAMPH enhanced working memory, attention, and information processing speed (Mahoney et al., 2011). This human study gave a trial injection of 30 mg MAMPH to the subject. If we assumed that the subject weighed 60 kg, this dose could be translated to approximately 1.36 mg for a rat weighing 300 g (Nair and Jacob, 2016). The present study used a lower dose of MAMPH (1 mg/kg) in rats than in the previous human study, meaning that MAMPH administrations used in the present study may enhance cognitive function and neuronal plasticity but will not cause cognitive decline or neurotoxicity.

As per our anticipatory results, the present data showed that MAMPH administrations elicit CTA learning and increase neural activity in c-Fos expression and neural plasticity in p-ERK expression in the neural substrates related to cognitive function. In the subareas of the mPFC (including the Cg1, PrL, and IL), MAMPH activated CTA learning and memory. Moreover, the DG of the hippocampus, which mediates spatial learning, elicited neural activity and neural plasticity after MAMPH administration. This evidence appears to support results from previous animal and human models. For example, low doses of MAMPH were shown to enhance LTP activity and spinal enlargement in the dentate granule cells of the DG (Baptista et al., 2016). In the passive avoidance conditioning test, rats with MAMPH injections were able to boost their memory, indicating that MAMPH may be a cognitive enhancer that could aid memory retention (Matsuoka et al., 1992). Low concentrations of MAMPH have been shown to increase dopamine transmission and dopamine firing rates, while higher concentrations of MAMPH have reduced dopamine D2 autoreceptor activity and decreased the peak amplitude of dopamine’s synaptic currents (Branch and Beckstead, 2012). In an *in vivo* study, a low dose of MAMPH exposure regulated beta-amyloid precursor protein-cleaving secretases and stimulated the soluble amyloid precursor protein alpha for improving cognitive functions in AD patients (Shukla et al., 2019).

In human models, the previous findings are similar to those in animal studies. MAMPH may ameliorate an AD patient’s numerous cognitive functions, including visuospatial perception, attention span, inhibition, working memory, long-term memory and learning, and neuropsychiatric symptoms for a specific condition. For example, a low dose or a one-off administration of MAMPH (Hart et al., 2012) could be used as a cognitive enhancer for treating AD symptoms (Shukla and Vincent, 2020). One-off administrations and relatively small concentrations of amphetamine (90, 95, 105 ng/ml) and MAMPH (72, 67, and 59 ng/ml) were found to enhance psychomotor function and perceptual speed in attention studies (Silber et al., 2006). Intermediate doses (12 and 25 mg/70 kg) of MAMPH, applied using intranasal administration, were shown to improve cognitive and psychomotor functions that decreased hit latency, increased maximum tracking speed for improving divided attention performance, and enhanced performance in a total attempt and correct in the digit-symbol-substitution task (Hart et al., 2008). Using 15 and 30 mg MAMPH administration by the intravenous way increased attention, concentration, and psychomotor performance (Johnson et al., 2005). In addition, a low dose of 10 mg MAMPH appeared to increase reaction time in a memory scanning task (Mohs et al., 1980). Psychostimulants (e.g., amphetamine and methylphenidate) can improve apathy symptoms in AD patients (Scherer et al., 2018). In summary, the MAMPH administrations may ameliorate cognitive impairments and neuropsychiatric symptoms and activate neuroactivity and synaptic plasticity. Administrating the specific doses of MAMPH over specific trials and durations could potentially treat AD symptoms in the future.

By contrast, MAMPH administrations can also induce c-Fos or p-ERK expression in the affected brain area- rewarding the NAc and rewarding and aversive the BLA. In this treatment regimen, MAMPH administrations caused drug addiction and negative emotional responses. These adverse effects of MAMPH should also be taken into consideration when developing treatment regimens for AD symptoms.

### Prelimbic Cortex Modulation and Methamphetamine Administrations

In the present study, the MAMPH administrations linked to PrL excitation with a low concentration of NMDA enhanced a single MAMPH administration-induced neural activity in c-Fos expression and neural plasticity in p-ERK expression in the PrL and IL of the mPFC. The results indicated that the excitation of the PrL neurons underlying MAMPH administrations upregulated c-Fos expression in neural activity and p-ERK expression in neural plasticity. The PrL may also play a role in enhancing MAMPH’s effect on neural activity and plasticity. The involvement of the PrL produces a combined effect for facilitating MAMPH-induced neural activity and plasticity in the mPFC—the area of the brain associated with cognition. Unfortunately, no research currently exists that examines the effect of the PrL in MAMPH’s neural activation and cognitive function in animal models. Our findings were associated with the viewpoint that the prefrontal cortex regulates various cognitive and executive functions, including working memory, inhibitory control, decision making, attention, spatial learning, and long-term memory (Dalley et al., 2004; Jobson et al., 2021). In light of this indirect viewpoint, the excitation of the PrL of the mPFC might be expected to enhance cognitive and executive processing in the frontal cortex, thereby facilitating MAMPH-induced neural activity and plasticity in the mPFC, particularly in the PrL and IL.

On the other hand, the PrL excitation inhibited CTA conditioned learning induced by MAMPH and plasma corticosterone levels, whereas the PrL lesion enhanced MAMPH-induced CTA learning and corticosterone levels in the plasma. The present study also indicated that the PrL plays a role in inhibitory control to blunt the aversive CTA learning and corticosterone secretions (Jobson et al., 2021).

The data also showed that the PrL has an inhibitory role for the DG. Regarding the DG, the PrL excitation inhibited the spatial learning brain area (the DG of the hippocampus) in neural activity for c-Fos expression or neural plasticity for p-ERK expression. Conversely, the PrL lesion enhanced neural activity or neural plasticity in the DG of the hippocampus. However, the PrL excitation also inhibited the BLA’s neural activity in c-Fos expression and neural plasticity in p-ERK expression, and the PrL lesion increased neural acidity in c-Fos expression and neural plasticity in p-ERK expression, indicating the PrL also plays a role of an inhibitory regulation for the BLA.

It is well known that the mPFC projects different brain areas to mediate distinct functions (Dalley et al., 2004). In the structural aspect, the mPFC interacts significantly with the cortical subregions, including the thalamus, NAc, amygdala, and hippocampus (Riga et al., 2014; Jobson et al., 2021). In the functional aspect, it remains unknown as to whether the mPFC (particularly the PrL) interacted with these subregions, and it always played an inhibitory control to the NAc, amygdala, and hippocampus. Alternatively, it may contribute to regulating the cortical subregions under certain conditions. This emerging issue should be investigated in future studies.

### Plasma in Corticosterone Levels and Stress Underlying the Methamphetamine Administrations

Whether or not MAMPH administration affects plasma corticosterone remains uncertain for the following reasons? For example, a high dose of MAMPH (10 mg/kg) caused an increase in plasma corticosterone levels and BDNF for neonatal rats during the postnatal days 11–15 (Grace et al., 2008). However, adolescent mice with acute MAMPH exposure exhibited increased locomotor activity and anxiety behavior, although their plasma corticosterone levels remained steady (Rud et al., 2016). After acute MAMPH administration, female (but not male) mice exhibited a prolonged increase in plasma corticosterone levels, indicating that the animal’s sex can affect plasma corticosterone levels after MAMPH administration (Zuloaga et al., 2014). Another study pointed out the importance of the time point, as MAMPH treatments increased plasma corticosterone levels between 1 and 72 h, with the peak point occurring during the first hour (Herring et al., 2008). The results showed that MAMPH administration elevates plasma corticosterone, which might also show that MAMPH enhances stress status. The data were consistent with the above viewpoint that MAMPH-elicited corticosterone levels in plasma and anxiety and aversive behaviors (Grace et al., 2008; Herring et al., 2008). Alternatively, the excitation or lesion of the PrL neurons decreased or increased MAMPH-induced plasma corticosterone levels in the present study. The PrL might also play executive and inhibitory roles in the regulation of MAMPH administration-induced stress effects. This viewpoint is seemingly consistent with the fact that the PrL of the mPFC inhibits neural activity within the amygdala (Laurent and Westbrook, 2008; Koenigs and Grafman, 2009). The mPFC-amygdala pathway conveys a negative valence of the emotion of the amygdala to the mPFC, meaning that the mPFC inhibits the negative emotional response from the amygdala. The balance of the mPFC-amygdala pathway is to maintain the individual’s psychological health (Peters et al., 2009; Kim et al., 2011). MAMPH may induce a stress effect and may release plasma corticosterone. However, the PrL’s modulation of the mPFC is essential for reducing the stress effect induced by MAMPH administrations.

### Experimental Limitations and Emerged Issues

The present study had some experimental limitations. First, the present study used healthy rats to test whether MAMPH administration affected conditioned learning in the mPFC (cognitive function), the NAc, the amygdala (the affected brain areas), or the hippocampus (the spatial learning area) by measuring plasma corticosterone levels; neural activity, assessed by measuring c-Fos expression; or neural plasticity, via assessed by measuring p-ERK expression. The study also clarified whether MAMPH administered to the PrL affected CTA, neural activity, or plasticity in the mPFC, NAc, amygdala, and hippocampus. Our study found that the short-term administration of MAMPH serves as a cognitive enhancer in a typical animal model of CTA (Yu et al., 2021); however, the model used in this study was not a typical animal model of AD. Repeating these tests in a suitable animal model of AD [e.g., a transgenic (Weishaupt et al., 2018) or a neurotoxic lesion-induced mouse model (Ando et al., 2002) of AD] and examining the effects on CTA under AD conditions remains necessary in future studies to determine whether these findings are applicable in the AD brain.

Second, this study only used rats aged between 6 and 8 weeks (1.5–2 months). Some studies have suggested that younger rats are suitable for AD animal models (El Tamer et al., 1992, 1996). For example, AD-related animal studies of reversible cholinergic changes have demonstrated that the intracerebroventricular administration of AF64A to induce cholinergic toxicity resulted in decreased ChAT activity and increased AChE activity in the hippocampus and septal regions of young rats (2, 4, and 12 months), whereas older rats, aged 22 months, showed no changes in ChAT and AChE activity (El Tamer et al., 1992, 1996). However, other AD studies have used older animals (Weishaupt et al., 2018). For example, in APP21 transgenic rats aged 19 months, working memory deficits were observed in strategy shifts and facilitated white matter inflammation (Weishaupt et al., 2018). Therefore, rats of different age groups should be tested in future experiments.

In the statistical analysis, Tukey’s HSD test was conducted after one-way ANOVA. According to statistical rules, Tukey’s HSD test was used for those analyses in which the sample sizes of different groups were equal; however, when the sample size is unequal, the Tukey’s HSD equation should be modified using the Tukey-Kramer procedure (Montgomery, 2013). Therefore, when analyses were performed on groups in which the sample size was not equal, the *post hoc* test was conducted using the Tukey-Kramer procedure.

Finally, this study only tested CTA conditioned learning. The CTA animal task is an aversively conditioned learning process; moreover, it is actually an implicit learning and memory task. Therefore, different learning and memory tasks also need to be investigated in order to determine whether MAMPH enhances cognitive performances in other implicit (e.g., reward conditioned learning) and explicit learning and memory tasks in the animal model.

## Conclusion

Using a low dose of 1 mg/kg MAMPH with three continuous administrations in the present study produced CTA conditioned learning, increases in plasma corticosterone levels, and enhancements in neural activity in c-Fos expression, and neural plasticity in p-ERK expression in the cognitive function areas (the mPFC, e.g., Cg1, PrL, and IL), the affected brain areas (the rewarding NAc and rewarding and aversive BLA), and the spatial learning and memory brain areas (the hippocampus’ CA3 and DG). Additionally, the excitation of the PrL neurons induced more enhancements for neural activity and neural plasticity in the PrL and IL of the mPFC’s subareas, indicating that a low dose of MAMPH in conjunction with the excitation of PrL neurons might lead to a higher level of neural activity and plasticity than a single MAMPH administration in ameliorating cognitive functions. Therefore, low doses of MAMPH and the modulation of PrL neurons might provide a possible treatment for certain symptoms of AD.

## Data Availability Statement

The datasets presented in this study can be found in online repositories. The names of the repository/repositories and accession number(s) can be found in the article/Supplementary Material.

## Ethics Statement

The animal study was reviewed and approved by the Fo Guang University Institutional Animal Care and Use Committee.

## Author Contributions

B-CS: methodology, validation, investigation, data curation, supervision, and funding acquisition. ZY-G: data curation and project administration. JW: formal analysis and project administration. C-NC: preparing figures and project administration. ABHH: data curation. ACWH: conceptualization, methodology, formal analysis, writing (original draft), writing (review and editing), and funding acquisition. All authors contributed to the article and approved the submitted version.

## Conflict of Interest

The authors declare that the research was conducted in the absence of any commercial or financial relationships that could be construed as a potential conflict of interest.

## Publisher’s Note

All claims expressed in this article are solely those of the authors and do not necessarily represent those of their affiliated organizations, or those of the publisher, the editors and the reviewers. Any product that may be evaluated in this article, or claim that may be made by its manufacturer, is not guaranteed or endorsed by the publisher.
