# Supplementary material for: Methamphetamine and Modulation Functionality of the Prelimbic Cortex for Developing a Possible Treatment of Alzheimer’s Disease in an Animal Model
Source: Front Aging Neurosci. 2021 Oct 20;13:751913. doi: 10.3389/fnagi.2021.751913 (PMC8564002; doi:10.3389/fnagi.2021.751913)
Supplement: Supplementary file 1 [file Data_Sheet_1.docx]

Supplementary Material

# Supplementary Data

All experimental data are uploaded to the website below.

<https://www.dropbox.com/sh/n35o7ui1bz7vxy7/AADb31ocnVjuOokFnaHu-cXKa?dl=0>

# Supplementary Figures and Tables

## Supplementary Figures

**
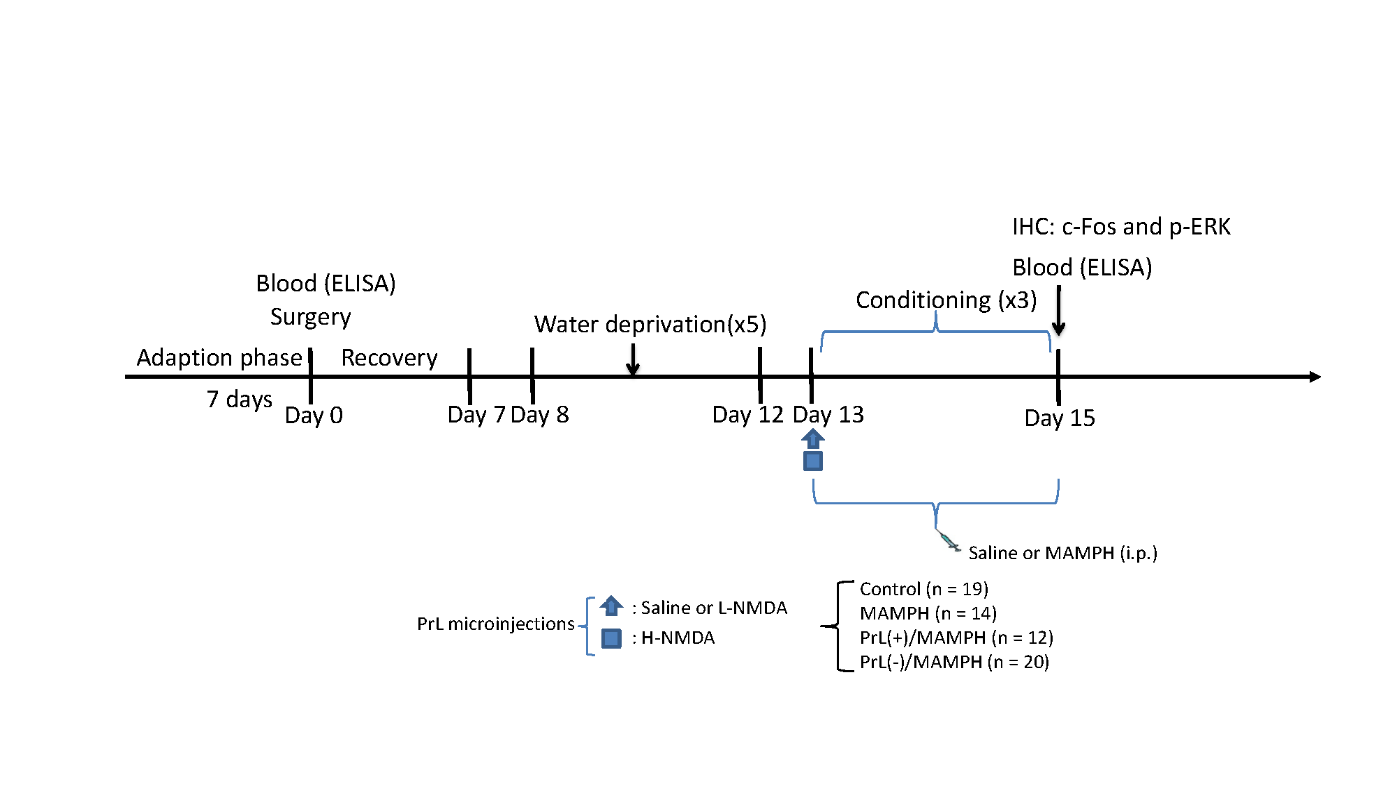
**

**Supplementary Figure 1.** Overview of the experimental procedures. The behavioral processes were shown in the adaptation, water deprivation, and conditioning phases. On Day 13, the low or high concentrations of NMDA or saline were microinjected in the PrL. On conditioning (Days 13-15), a low dose of MAMPH (1 mg/kg) or saline was paired with 0.1% saccharin solution to form CTA for three sessions for the Control, MAMPH, PrL(+)/MAMPH, and PrL(-)/MAMPH groups. Then, the immunohistochemical staining with c-Fos or p-ERK expression was performed to label the selected neural substrates after behavioral tests. In addition, the blood was collected to assess corticosterone in plasma using the ELISA approach on the last day of the adaptation phase on Day 0 and the final session of the conditioning phase on Day 15. PrL(+): the low concentration of NMDA microinjections in the PrL excites the PrL neurons; PrL(-): the high concentration of NMDA microinjections in the PrL excites the PrL neurons.

| **Supplementary Table 1.** Summary for modulation of the PrL neurons and methamphetamine administrations in a variety of tests including CTA learning, plasma corticosterone levels, neural activity in c-Fos expression, and neural plasticity in p-ERK expression. | | | | |
| --- | --- | --- | --- | --- |
|  | CTA learning | Plasma corticosterone levels | Neural activity labeling c-Fos expression | Neural plasticity labeling p-ERK expression |
| Control group | --- | --- | --- | --- |
| MAMPH group | ↑ | ↑ | Cg1↑, PrL↑, IL↑  NAc↑, BLA↑, DG↑ | PrL↑, IL↑,  BLA↑, DG↑ |
| PrL(+)/MAMPH group | ↓ | ↓ | Cg1↓, PrL↑, IL↑  BLA↓, DG↓ | Cg1↑, PrL↑, IL↑  BLA↓, DG↓ |
| PrL(-)/MAMPH group | ↑ | ↑ | Cg1↓, PrL↓  IL↓, DG↑ | PrL↓, IL↓  BLA↑, DG↑ |

Note: MAMPH: methamphetamine; PrL(+)/MAMPH: the excitation of the prelimbic cortex microinjection with a low concentration of NMDA prior to methamphetamine intraperitoneal injections; PrL(-)/MAMPH: the lesion of the prelimbic cortex microinjection with a high concentration of NMDA prior to methamphetamine intraperitoneal injections; ↑: increase ; ↓: decrease; Cg1: cingulate cortex area 1; PrL: prelimbic cortex; IL: infralimbic cortex; NAc: nucleus accumbens; BLA: basolateral amygdala; DG: dentate gyrus. The data of the MAMPH group was compared to the Control group; The data of the PrL(+)/MAMPH and PrL(-)/MAMPH groups were compared to the MAMPH group.
